# Beneficial Effect of Sugar Osmolytes on the Refolding of Guanidine Hydrochloride-Denatured Trehalose-6-phosphate Hydrolase from *Bacillus licheniformis*

**DOI:** 10.1155/2015/806847

**Published:** 2015-01-13

**Authors:** Jiau-Hua Chen, Meng-Chun Chi, Min-Guan Lin, Long-Liu Lin, Tzu-Fan Wang

**Affiliations:** ^1^Department of Food Science and Technology, Chia-Nan University of Pharmacy and Science, Tainan City 71710, Taiwan; ^2^Department of Applied Chemistry, National Chiayi University, 300 Syuefu Road, Chiayi City 60004, Taiwan; ^3^Department of Chemistry, National Cheng Kung University, Tainan City 701, Taiwan

## Abstract

The influence of three sugar osmolytes on the refolding of guanidine hydrochloride- (GdnHCl-) denatured trehalose-6-phosphate hydrolase of *Bacillus licheniformis* (*Bl*TreA) was studied by circular dichroism (CD) spectra, fluorescence emission spectra, and the recovery of enzymatic activity. These experimental results clearly indicated that sorbitol, sucrose, and trehalose at a concentration of 0.75 M improved the refolding yields of GdnHCl-denatured  *Bl*TreA, probably due to the fact that these sugars favored the formation of tertiary architectures. Far-UV CD measurements demonstrated the ability of sugar osmolytes to shift the secondary structure of GdnHCl-denatured enzyme towards near-native conformations. ANS fluorescence intensity measurements revealed a reduction of exposed hydrophobic surfaces upon the treatment of denatured enzyme with sugar osmolytes. These observations suggest that sugar osmolytes possibly play a chaperone role in the refolding of chemically denatured *Bl*TreA.

## 1. Introduction

Protein folding is a biological process by which the primary structure of proteins folds into defined architectures to gain relevant functions. Despite numerous advances in the last five decades, the elucidation of the molecular mechanism of protein folding from a disordered polypeptide to the native state remains one of the major challenges in the field of protein chemistry [1, 2]. However, detailed reviews of the extensive structural and biochemical studies have demonstrated that molecular chaperones play an important role in protein folding* in vivo* as well as* in vitro* [3–5]. A molecular chaperone transiently binds and stabilizes the unstable conformation of a specific protein, thereby facilitating protein folding and preventing it from misfolding and aggregation [6, 7].

Proteins are dynamic entities that are in constant interaction with their environments. Several components of the protein environment can affect the folding landscape [8]. They include solvents [9], crowding agents [10], osmolytes [11], and small-molecule and macromolecular ligands [12–14]. Naturally occurring osmolytes are low-molecular weight compounds that are utilized by biological systems as chemical chaperones to counteract deleterious effects generated from extreme physical conditions, such as high osmotic and hydrostatic pressures [15, 16], dehydration [17], and high or low temperatures [16, 18]. They are one of the most potent stabilizers for many proteins and are capable of protecting them from denaturation or aggregation [19–21]. Naturally occurring osmolytes have also been found to modulate activity of molecular chaperones (heat-shock proteins) probably because of the promotion of local refolding within the chaperone protein molecules, suggesting a link between the chemical and molecular chaperones in regulation of protein folding* in vivo* [22]. In this regard, it is logical that cells regulate many biological processes such as protein folding, protein disaggregation, and protein–protein interactions* via* accumulation of specific osmolytes.

Understanding the relationship between structural and functional connectivity is of crucial importance in the practical application of enzymes. Denaturation and renaturation are thermodynamic processes, involving a change in free energy and large changes in conformation between the denatured and the native states [23]. Misfolding and aggregation pose a serious problem in the production and use of recombinant proteins. Aggregation may be due to the association of hydrophobic surfaces that are exposed during the refolding process [24]. A strategy to prevent aggregation by interfering with intermolecular hydrophobic interactions is to use sugar osmolytes that are relatively inexpensive and easy to remove once folding is complete. Sugar osmolytes have proven to be effective folding aids with several proteins [25–28].

Family GH13 is the major glycoside hydrolase family acting on substrates containing *α*-glucoside linkages. As a member of family GH13, trehalose-6-phosphate hydrolase (TreA) cleaves the *α*,*α*-1,1-glycosidic linkage of trehalose-6-phosphate to produce glucose and glucose-6-phosphate and plays a role in bacterial trehalose metabolism [29]. Recently, we have characterized the TreA protein from* Bacillus licheniformis* (*Bl*TreA) at the molecular level [30]. The recombinant enzyme starts to unfold beyond ~0.14 M guanidine hydrochloride (GdnHCl) and reaches the unfolded intermediates, [GdnHCl]_0.5,N–I_ and [GdnHCl]_0.5,I–U_, at 1.02 and 2.24 M, respectively. Given that the refolding of GdnHCl-denatured enzymes has been fairly well studied [31–34], we perform the influence of sugar osmolytes on the refolding of GdnHCl-denatured* Bl*TreA. The present investigation indicates that the tested sugar osmolytes probably act as a chemical chaperone to facilitate the formation of secondary and tertiary* Bl*TreA structures. Our study also suggests one strategy to enhance the percentage of correct protein refolding through the addition of sugar osmolytes into the refolding buffer.

## 2. Materials and Methods

### 2.1. Materials

Sorbitol, sucrose, and trehalose were acquired from Wako Pure Chemicals (Tokyo, Japan). Guanidine hydrochloride (GdnHCl), 1-anilino-8-naphthalenesulfonate (ANS),* p*-nitrophenyl-*α*-D-glucopyranoside (*p*NPG), and* p*-nitrophenol (*p*NP) were obtained from Sigma-Aldrich Chemicals (St. Louis, MO, USA). All other chemicals used were of analytical grade or the equivalent.

### 2.2. Enzyme Purification, Activity Assay, and Determination of Protein Concentration

Purification of* Bl*TreA from* Escherichia coli* M15 (pQE-*Bl*TreA) was according to the procedure described previously [30]. TreA activity was assayed by mixing a 0.5 mL aliquot of enzyme in 50 mM Hepes-NaOH buffer (pH 8.0) with 0.5 mL of 10 mM* p*NPG and 200 mM NaCl and subsequently incubated the reaction mixture at 30°C for 10 min. The hydrolysis of* p*NPG was determined by measuring the absorbance of heat-treated sample at 410 nm. One unit of* Bl*TreA activity is defined as the amount of enzyme that produces 1 *μ*mol of* p*NP per min at 30°C.

Protein concentrations were determined using the Bradford reagent (Bio-Rad) and bovine serum albumin as a standard protein.

### 2.3. Denaturation/Renaturation Studies

All experiments on denaturation and renaturation were carried out in 50 mM Hepes-NaOH buffer (pH 8.0). For enzyme denaturation, 0.24 mM* Bl*TreA was treated with 6 M GdnHCl and allowed to stand for 12 h. Aliquots (5 *μ*L) were diluted with 50 mM Hepes-NaOH buffer (pH 8.0) to give final concentrations of 2.4 *μ*M* Bl*TreA and 60 mM GdnHCl. After 30 min, 60 *μ*L of osmolytes or 60 *μ*L of ddH_2_O was added to bring the final concentrations of 3.4 *μ*M* Bl*TreA, 50.4 mM GdnHCl, and 0.30–1.25 M sugars. The samples were incubated at 4°C for 12 h and then assayed for TreA activity.

### 2.4. Fluorescence and Far-UV Circular Dichroism (CD) Studies

The refolding of GdnHCl-denatured* Bl*TreA was studied by observing its fluorescence spectra, far-UV CD spectra, and ANS fluorescence spectra. Fluorescent intensity measurements were carried out on a JASCO FP-6500 spectrophotometer equipped with a thermostatically controlled cuvette compartment. Emission spectra were recorded from 300 to 450 nm with an excitation wavelength of 280 nm.

The secondary structural changes of refolding of GdnHCl-denatured* Bl*TreA were measured by recording far-UV CD spectra on a JASCO model J-815 spectropolarimeter from 250 to 190 nm in cuvettes with a 1.0 nm bandwidth, 0.1 nm resolution, 0.1 cm path length, 1.0 s response time, and 100 nm/min scanning speed. Each scanning was repeated ten times to ensure a good noise ratio. The obtained data were corrected for the buffer effect and the experimental results were expressed as molar ellipticity [*θ*] in the units of degree·cm^2^·decimol^−1^ according to the following:

(1) $$\begin{array}{c} \left[\theta \right]=\frac{\theta }{10\times C\times l}, \end{array}$$

where *l* is the light path length in centimeter, *C* is the molar concentration of protein in mol/L, and *θ* represents the observed ellipticity in degrees at a given wavelength.

For determining of the binding of ANS to GdnHCl-denatured* Bl*TreA at different concentrations of sugar osmolytes, ANS was added to a final concentration of 40 *μ*M to the protein samples that had been preincubated for 12 h in the presence of sugar osmolytes, and then ANS fluorescence spectra were recorded from 400 to 600 nm with an excitation wavelength of 350 nm.

## 3. Results and Discussion

**Figure 1:**
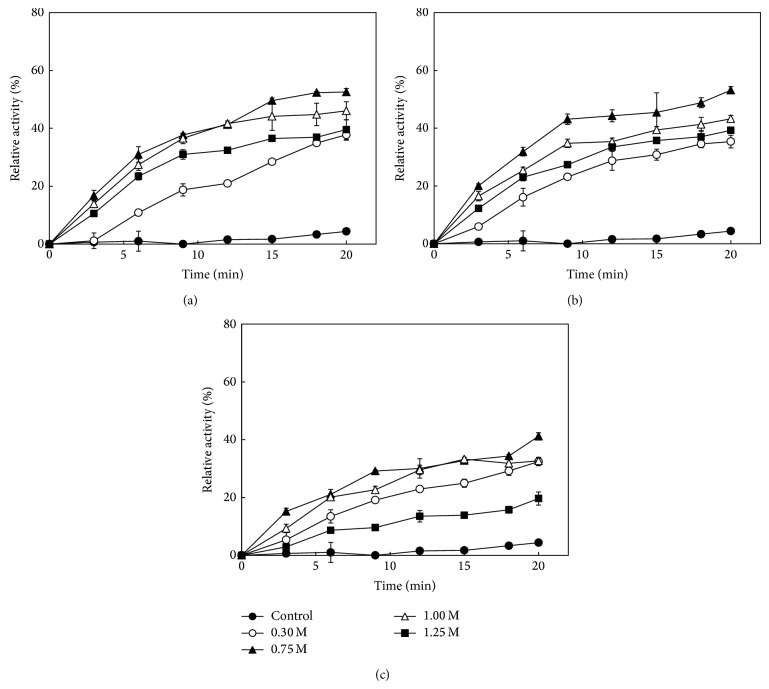
Reactivation of GdnHCl-denatured* Bl*TreA in the presence of various concentrations of sugar osmolytes. Reactivation was initiated by diluting the unfolded enzyme into the standard buffer (50 mM Hepes-NaOH buffer, pH 8.0) in the absence (control) and presence of various concentrations of sugars, including sorbitol (a), sucrose (b), and trehalose (c). TreA activity was measured at the indicated times and the enzymatic activity of native enzyme was taken as 100%.

**Figure 2:**
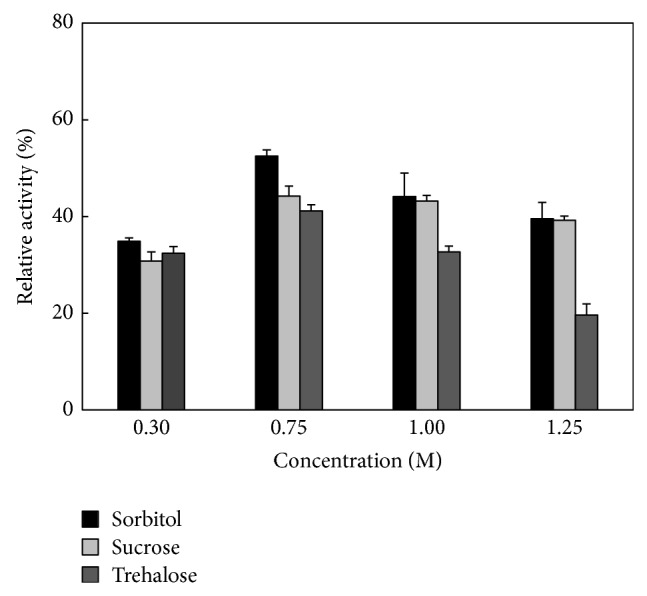
Final yield of* Bl*TreA in the absence and presence of different osmolytes. The experimental data is derived from Figure 1.

To evaluate the reactivation of denatured* Bl*TreA, the protein samples were diluted with 50 mM Hepes-NaOH buffer (pH 8.0) to a final concentration of 5.0 *μ*M. As compared to the self-renatured* Bl*TreA, the reactivation rate of GdnHCl-denatured enzyme was highest by adding 0.75 M of sorbitol, sucrose, and trehalose into the renaturation system (Figure 1). However, the TreA activity gradually decreased upon the addition of 1.25 M sugar osmolytes. The final reactivation yields are shown in Figure 2. The experimental data indicate that sorbitol was more effective than any of the other two sugars for the reactivation of GdnHCl-denatured* Bl*TreA. Also, it is worth to note that all these osmolytes affected the* Bl*TreA reactivation in a concentration dependent manner.

**Figure 3:**
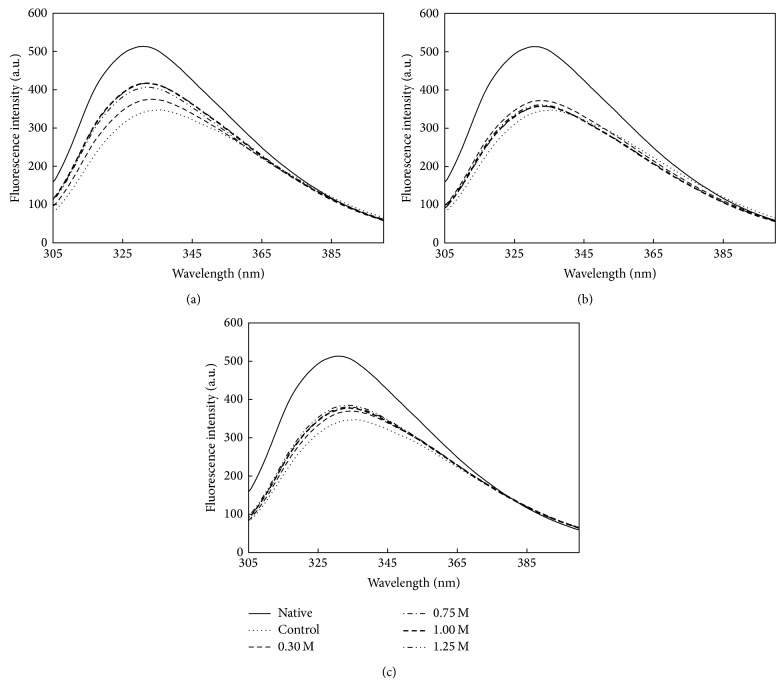
Intrinsic fluorescence emission spectra of refolded* Bl*TreA in the presence of various concentrations of sugar osmolytes. Refolding occurred by diluting the unfolded enzyme into the standard buffer (50 mM Hepes-NaOH buffer, pH 8.0) in the absence (control) and presence of various concentrations of sugars, including sorbitol (a), sucrose (b), and trehalose (c). The native and unfolded enzymes were used as positive and negative controls.

The reversibility of unfolding was studied using various parameters including the exposure of tryptophan residues, loss of secondary structure, and the exposure of hydrophobic surface. Tryptophan fluorescence emission spectrum of native* Bl*TreA was characterized by a peak centered at 331.4 nm. The fluorescence markedly changes when the protein was in the unfolded state, with a shift in the emission maximum to 355.6 nm [30]. When GdnHCl-denatured* Bl*TreA was diluted with the refolding buffer containing various amounts of sorbitol, sucrose, and trehalose, there was a rapid return of the fluorescence wavelength maximum to 336.2 nm (Figure 3). The blue shift of the maximum of fluorescence emission from the unfolded state suggests that most of the Trp residues have recovered the environment as they have in the native state.

**Figure 4:**
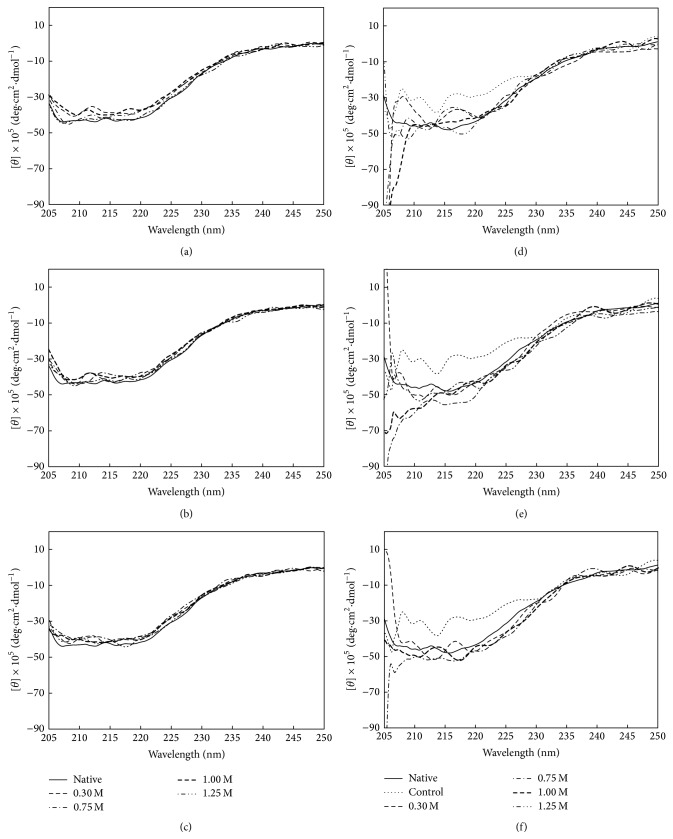
Far-UV CD spectra of native (a–c) and refolded (d–f)* Bl*TreA in the presence of various concentrations of sugar osmolytes. Refolding occurred by diluting the unfolded enzyme into the standard buffer (50 mM Hepes-NaOH buffer, pH 8.0) in the absence (control) and presence of various concentrations of sugars, including sorbitol (d), sucrose (e), and trehalose (f). The native and unfolded enzymes were used as positive and negative controls.

The enzyme was mostly in a refolded form as detected by fluorometric experiment; however, the corresponding recovery in TreA activity was less than 56%. Far-UV CD spectra of osmolyte-treated samples were, therefore, measured (Figure 4) and deconvolution of the spectra for secondary structure amount was subsequently performed using CDNN software [35]. Because of the presence of 50.4 mM GdnHCl, the spectra were presented only in the range of 250–205 nm. It was shown that native* Bl*TreA exhibited a strong positive maximum at 192 nm (data not shown) and two negative minima at 208 nm and 222, characteristic of the high *α*-helix content of the enzyme. The spectral properties of native* Bl*TreA were preserved even after the addition of sugar osmolytes into the enzyme solution (Figure 4). As a control, the GdnHCl-denatured enzyme did not show the typical CD spectra of *α*-helix. Refolding of GdnHCl-denatured* Bl*TreA in the presence of different concentrations of sugar osmolytes resulted in some recovery of the ellipticity of the protein (Figure 4). The estimates of the secondary content showed that the *α*-helix and *β*-strand content of the native enzyme were 45% and 27%, respectively, and those of the partially folded structure were 37–50% and 23–31%, respectively. These results indicate that the intermediate is partially folded in the presence of sugar osmolytes. This situation may be responsible for the incomplete recovery of the TreA activity of the refolded enzyme.

**Figure 5:**
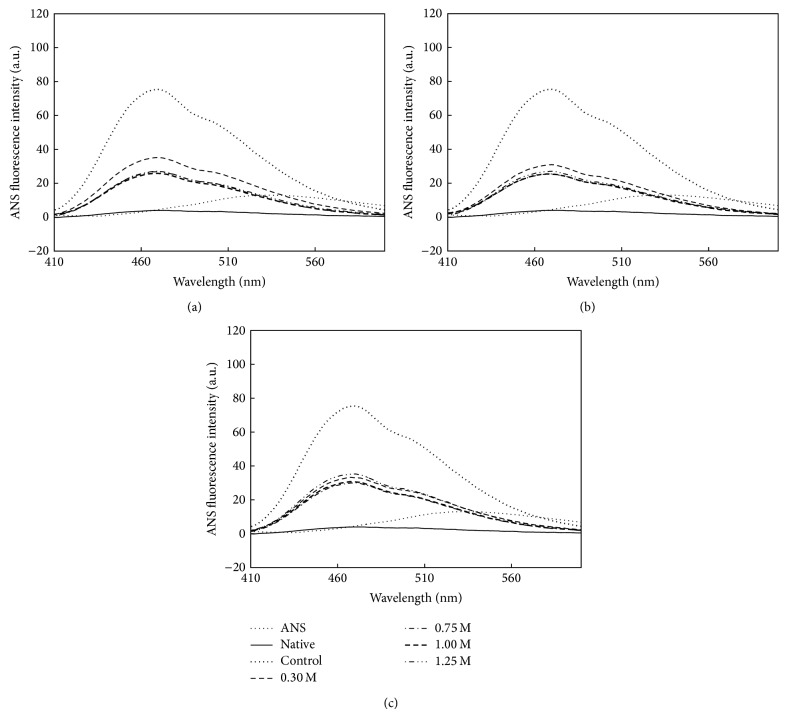
ANS fluorescence emission spectra of refolded* Bl*TreA in the presence of various concentrations of sugar osmolytes. Refolding occurred by diluting the unfolded enzyme into the standard buffer (50 mM Hepes-NaOH buffer, pH 8.0) in the absence (control) and presence of various concentrations of sugars, including sorbitol (a), sucrose (b), and trehalose (c). The native and unfolded enzymes were used as positive and negative controls.

The hydrophobic fluorescent dye ANS is widely used to probe the exposure of the hydrophobic region upon protein unfolding. The binding of ANS to hydrophobic regions of proteins results in a profound enhancement of ANS fluorescence intensity and a significant blue-shift of the maximum wavelength. ANS fluorescence intensity measurements of* Bl*TreA refolding in the presence of various amounts of sugar osmolytes were, therefore, employed to investigate the exposure of the hydrophobic groups. As shown in Figure 5, the exposure of hydrophobic surface was markedly reduced upon the addition of sugar osmolytes. These results indicate that* Bl*TreA possess hydrophobic region that is buried in the native state but is exposed under the unfolded state. It also reflects that sugar osmolytes do help the denatured enzyme to refold into the correct native state.

For many proteins, compact conformations are known to accumulate in advance of the rate-limiting step in folding [1]. Disappearance of the hydrophobic clusters and the subsequent tight packing of the performed secondary structure are the rate-limiting steps of the folding process [36]. A primary driving force for protein folding involves the removal of nonpolar side chains from solvent exposure. Interactions with the aqueous solvent, known as the hydrophobic effect, lead to residues with nonpolar side chains typically being buried in the interior of a protein. In this regard, polypeptide-solvent interactions probably have a major impact on the refolding outcome [37]. As shown in Table 1, our experimental results confirm this opinion and show that a suitable concentration of sugar osmolytes is necessary for the denatured* Bl*TreA to acquire higher refolding yield and functional conformation.

Protein folding is a reversible process in which osmolytes push the folding equilibrium towards natively folded conformations by raising the free energy of the unfolded state [38]. As osmolytes predominantly affect the protein backbone, the balance between osmolyte-backbone interactions and amino acid side chain-solvent interactions guides protein folding [38]. It is well known that the stabilizing osmolytes are preferentially excluded from the intermediate vicinity of the protein surface through a solvophobic interaction between peptide backbone and side chain on the protein surface and the protecting osmolytes [39]. This tendency of osmolytes to be excluded from the protein surface forces polypeptides to adopt a folded conformation with a minimum possible exposed surface area [40]. It has been shown clearly that sorbitol and sucrose, acting as protective osmolytes, are preferentially excluded from the protein surface, increasing the latter's free energy [38]. This leads to thermodynamic stabilization of the protein due to the fact that the unfolded state becomes less favorable in the presence of sugar osmolytes [38, 41, 42]. In contrast to protective osmolytes, protein unfolding by the classical denaturants, such as urea and GdnHCl, has long been considered to arise because of the favorable interactions of the chemical agents with the normally buried segments of a protein [43]. The basis of biomolecular interactions for destabilization by these denaturants has been generally attributed to direct ligand binding with the protein surface or the influence of the denaturants on the structure and dynamic of water molecules [44, 45]. In our case, sorbitol, sucrose, and trehalose were found to offset the unfolding of GdnHCl on* Bl*TreA (Figures 1–5). It is widely argued that the refolding ability of organic osmolytes does arise primarily from the destabilization of the unfolded state of proteins upon osmolyte addition [46–48]. Thus, the beneficial effect of these sugars on the GdnHCl-denatured* Bl*TreA is likely due to destabilization of the unfolded state of the enzyme by sugar osmolytes. Timasheff and coworkers had also demonstrated a similar mechanism to explain the protective effect of osmolytes [40].

Compatible osmolytes increase protein stability against denaturation with little or no effect on their function under native conditions [49]. Representatives of this class include certain amino acids (e.g., proline and glycine) and polyols (e.g., trehalose, sucrose, and sorbitol). Although compatible osmolytes are largely accumulated to stabilize protein and enzyme systems, nature has not ignored the use of protein destabilizing osmolytes to act as efficient osmoprotectants. The metabolic waste, urea, and many other osmolytes (e.g., arginine, histidine, and lysine) are very good osmoprotectant [49]. Urea is a well-known chaotropic agent that disrupts the noncovalent interactions responsible for the globular structure of proteins [44]. In order to counteract the denaturing effects of urea on proteins* in vivo*, organisms or cells produce protective osmolytes, such as TMAO [50], betaine [51], and polyols [49], to stabilize protein structure and to maintain its activity in the presence of high levels of urea. No literature to date has indicated that organisms or cells utilize another chaotropic agent GdnHCl as an osmoprotectant. However, this chaotropic agent is a commonly used protein denaturant for the unfolding/refolding experiments [52]. Giving that the GdnHCl-induced protein denaturation can be counteracted by polyol-type osmolytes [27, 28, 33, 34, 41], our experimental data thus provide more insight into osmolyte-induced protein folding, and the findings are expected to facilitate the practical use of these sugars on the recovery of recombinant proteins from inclusion bodies.

Introduction of sugar osmolytes as excipients into protein solutions has been shown to affect their molecular properties in many ways. The general observations are that polyol-type osmolytes prevent the loss of enzymatic activity, inhibit irreversible aggregation of macromolecules, and protect proteins against thermal and chemical denaturation [49]. Despite the fact that widespread efforts have been made, the exact mechanism of the modulation of the chemical potential of proteins by sugar osmolytes is not well understood and hence, as a consequence, researchers do not dare to evaluate cosolvents other than a few selected ones for protein formulation. Our studies explore the present state of knowledge in regard to the beneficial effect of sugar osmolytes on the refolding of chemically denatured proteins. The experimental results can probably guide protein formulation with a better understanding of the selection criteria for the sugar osmolytes.

In conclusion, the potential of protective osmolytes and/or chemical chaperones as protein stabilizers has been further extended by the study of refolding of GdnHCl-denatured* Bl*TreA. Designing and developing formulations for protein preservation, particularly in the liquid state, would need the extensive understanding of the protective effect of sugar osmolytes on a wide variety of proteins. Based on our observations, sorbitol exerts a powerful ability in the refolding of GdnHCl-denatured enzyme. Therefore, this sugar might be useful for the design of new stabilizing and protective studies on the novel enzymes produced by the same* Bacillus* species.

## Figures and Tables

**Table 1:** Effect of sugar osmolytes on the refolding of denatured *Bl*TreA at 25°C.

Osmolyte concentration (M)	Sorbitol				Sucrose				Trehalose			
	I	II	III	IV	I	II	III	IV	I	II	III	IV
0.00	100	513.5	330.7	4.0	100	513.5	330.7	4.0	100	513.5	330.7	4.0
0.30	50.3	374.9	333.5	35.3	48.8	372.6	332.7	31.0	49.30	369.6	334.2	33.3
0.75	61.5	416.3	331.5	27.1	55.5	357.4	333.7	25.5	51.60	380.8	333.9	30.0
1.00	57.6	417.2	332.1	25.9	54.5	357.9	332.6	25.5	47.88	377.6	334.1	30.7
1.25	51.8	406.7	332.3	26.8	49.4	361.2	332.9	27.1	33.10	384.7	333.8	35.3

I: final refolding yield of *Bl*TreA; II and III (nm): maximum intensity and peak position of the intrinsic fluorescence emission spectra of the refolded *Bl*TreA in the presence of sugar osmolytes; IV: ANS fluorescence intensity of the refolded *Bl*TreA in the presence of sugar osmolytes.
